# Supplementary material for: Role of Myeloid Cell Glucose Transporter 1 in the Host Response During Pneumonia Caused by Streptococcus pneumoniae
Source: Int J Mol Sci. 2025 Oct 28;26(21):10461. doi: 10.3390/ijms262110461 (PMC12609635; doi:10.3390/ijms262110461)
Supplement: Supplementary file 1 [file ijms-26-10461-s001.zip › ijms-3905916-supplementary.pdf]

Role of myeloid cell glucose transporter 1 in the host response during pneumonia caused by *Streptococcus pneumoniae*

Liza Pereverzeva, Valentine Léopold, Anno Saris, Alex R. Schuurman, Joe M. Butler, Tom D.Y. Reijnders, Joris J.T.H. Roelofs, Daniël R. Faber, W. Joost Wiersinga, Cornelis van 't Veer, Alex F. de Vos and Tom van der Poll

Supplementary Figures and Tables

Figure S1. Gating strategy for monocytes and neutrophils and representative GLUT1, GLUT3 and 2-NBDG histograms

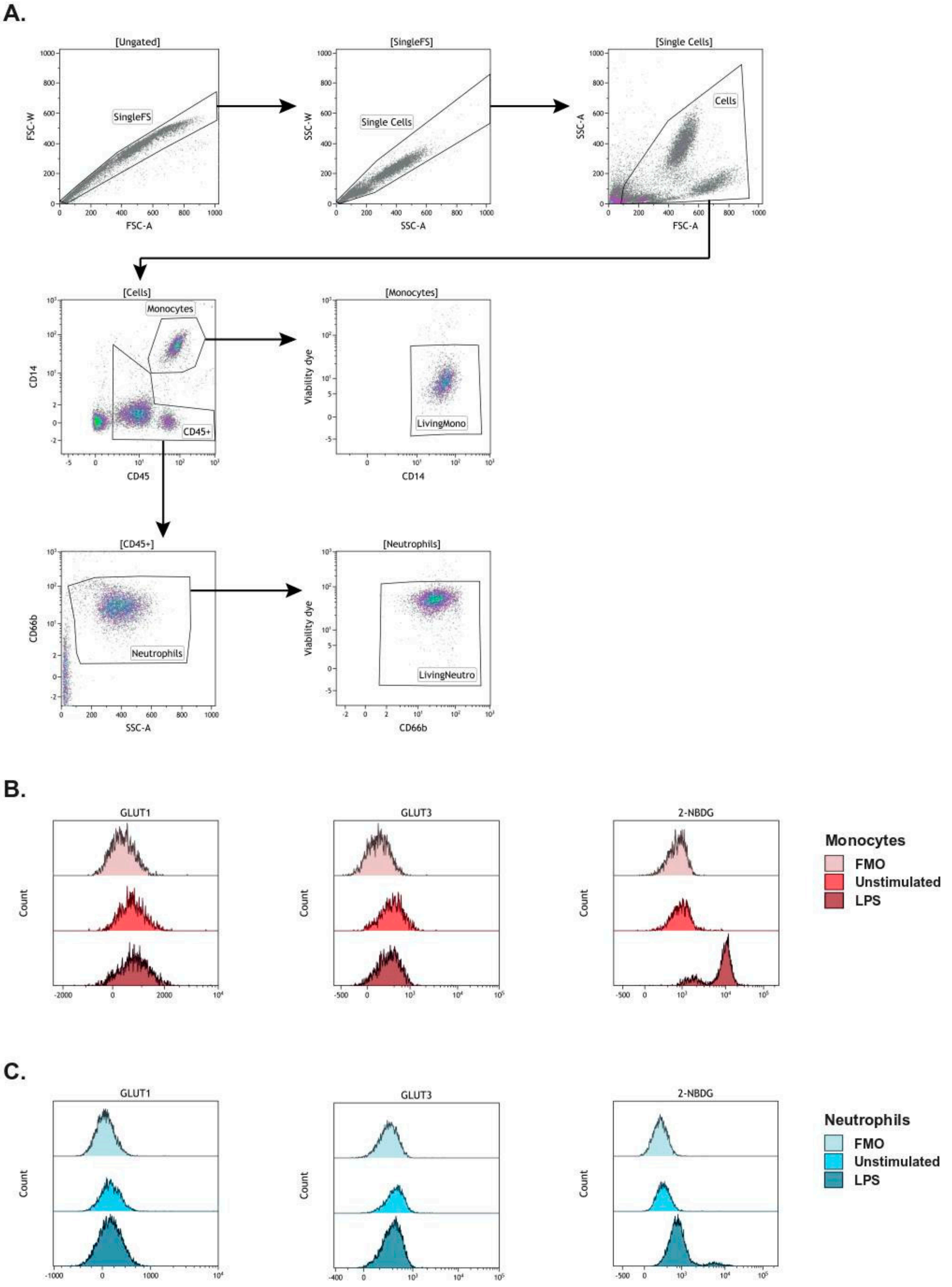

Gating strategy for monocytes (CD45+/CD14+) and neutrophils (CD45+/CD66b+) from whole blood (A). Representative histograms for GLUT1 and GLUT3 expression, and 2-NBDG uptake in unstained (fluorescent minus one (FMO)), unstimulated and LPS-stimulated monocytes (B) and neutrophils (C).

Figure S2. Pathology of lungs after *S. pneumoniae* infection

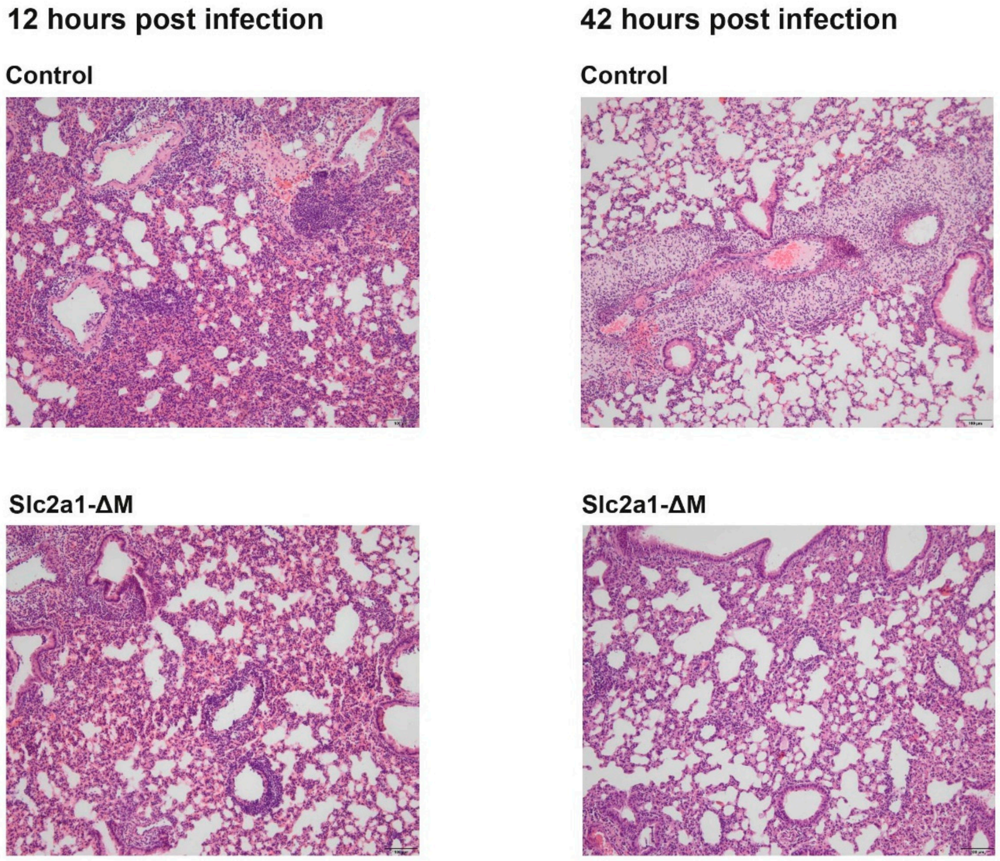

Representative photographs of haematoxylin and eosin (H&E)-stained tissue sections of infected lungs at 12 and 42 hours after inoculation with *S. pneumoniae* in *Slc2a1*-ΔM mice and littermate controls. Original magnification 10x

Figure S3. Bacterial dissemination after *S. pneumoniae* infection

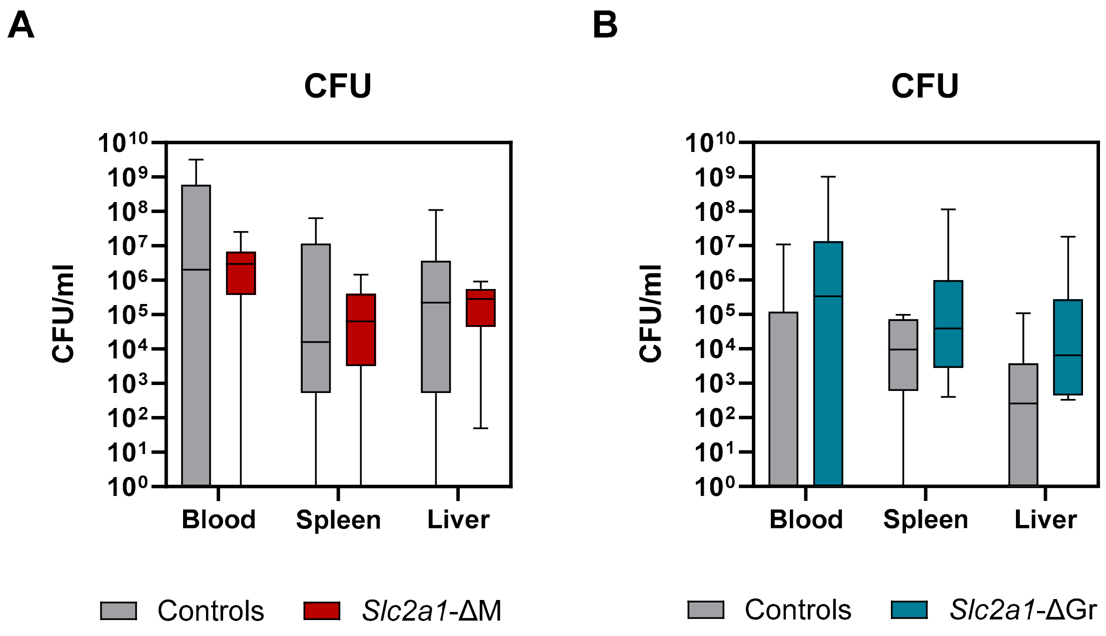

Bacterial counts (CFUs per milliliter) in blood, spleen and liver of myeloid cell-specific GLUT1 deficient (*Slc2a1*-ΔM) mice and littermate controls (A) and granulocyte-specific GLUT1 deficient (*Slc2a1*-ΔGr) mice and littermate controls (B) after intranasal inoculation with approximately $2x10^6$ CFUs of *S. pneumoniae* D39 analyzed 42 hours (A) and 40 hours later (B). All comparisons were non-significant (P >0.05).

Table S1: Clinical characteristics and disease course in cohort with gene expression of GLUTs in monocytes (1A), in neutrophils (1B), and with membrane expression of GLUTs and 2-NBDG uptake in monocytes and neutrophils (1C);

Table S1A. Clinical characteristics and disease course in cohort with gene expression of GLUTs in monocytes.

	CAP n=76	Controls n=42	*P* value
**DEMOGRAPHICS**			
Age (years)	70.2 (13.5)	69.4 (8.49)	0.69
Gender (male)	43 (56.6)	24 (57.1)	>0.99
BMI	27.1 (5.44)	27.8 (5.37)	0.51
**COMORBIDITIES**			
Asthma	6 (7.9)	1 (2.4)	0.42
Chronic obstructive pulmonary disease	25 (32.9)	4 (9.5)	0.007
Diabetes mellitus, type 1	1 (1.3)	2 (4.8)	0.29
Diabetes mellitus, type 2	20 (26.3)	2 (4.8)	0.003
Hypertension	34 (44.7)	21 (50.0)	0.70
Myocardial infarction	18 (23.7)	5 (11.9)	0.15
Stroke	5 (6.6)	0 (0.0)	0.16
Chronic kidney disease	7 (9.2)	1 (2.4)	0.26
**CHRONIC MEDICATION**			
Oral antidiabetics	16 (21.1)	2 (4.8)	0.018
Insulin	11 (14.5)	3 (7.1)	0.37
**LABORATORY TESTS**			
Thrombocytes (x10^9/L)	227 [57, 693]	NA	NA
Leukocytes (x10^9/L)	12.1 [2.40, 31.9]	NA	NA
Neutrophils (x10^9/L)	9.81 [1.62, 28.9]	NA	NA
Lymphocytes (x10^9/L)	1.09 [0.09, 25.0]	NA	NA
C-reactive protein (mg/L)	110 [2.90, 580]	NA	NA
**DISEASE SEVERITY**			
Pneumonia Severity Index	4.00 [1.00, 5.00]	NA	NA
CURB-65	2.00 [0.00, 4.00]	NA	NA
qSOFA	1.00 [0.00, 2.00]	NA	NA
**DISEASE COURSE**			
Duration of symptoms prior to admission (days)	4.00 [0.00, 40.0]	NA	NA
Hospital length of stay (days)	5.00 [0.00, 53.0]	NA	NA
28-day mortality	0 (0.0)	NA	NA

Continuous data are presented as mean (standard deviation) or median [interquartile range], and compared using a two-sided t-test or two-sided Wilcoxon rank-sum test, respectively. Categorical data are presented as count (percentage) and compared using Fisher's exact test. ACE = Angiotensin-converting enzyme, AT = Angiotensin, CAP = community-acquired pneumonia; CURB-65 = confusion, blood urea nitrogen, respiratory rate, blood pressure, age 65 or older; NA = not available or applicable, qSOFA = quick sequential organ failure assessment score.

Table S1B. Clinical characteristics and disease course in cohort with gene expression of GLUTs in neutrophils.

	CAP n=35	Controls n=13	*P* value
**DEMOGRAPHICS**			
Age (years)	69.3 (14.0)	69.2 (10.9)	0.97
Gender (male)	22 (62.9)	7 (53.8)	0.74
BMI	27.0 (5.35)	27.9 (3.79)	0.52
**COMORBIDITIES**			
Asthma	2 (5.7)	0 (0.0)	>0.99
Chronic obstructive pulmonary disease	12 (34.3)	2 (15.4)	0.29
Diabetes mellitus, type 1	1 (2.9)	1 (7.7)	0.47
Diabetes mellitus, type 2	10 (28.6)	2 (15.4)	0.47
Hypertension	14 (40.0)	8 (61.5)	0.21
Myocardial infarction	7 (20.0)	1 (7.7)	0.42
Stroke	4 (11.4)	0 (0.0)	0.56
Chronic kidney disease	5 (14.3)	1 (7.7)	>0.99
**CHRONIC MEDICATION**			
Oral antidiabetics	6 (17.1)	2 (15.4)	>0.99
Insulin	6 (17.1)	2 (15.4)	>0.99
**LABORATORY TESTS**			
Thrombocytes ($x10^9$/L)	232 [57.0, 693.0]	NA	NA
Leukocytes ($x10^9$/L)	12.2 [2.40, 27.4]	NA	NA
Neutrophils ($x10^9$/L)	9.29 [1.62, 24.6]	NA	NA
Lymphocytes ($x10^9$/L)	1.00 [0.09, 3.70]	NA	NA
C-reactive protein (mg/L)	115.0 [2.90, 580]	NA	NA
**DISEASE SEVERITY**			
Pneumonia Severity Index	4.00 [1.00, 5.00]	NA	NA
CURB-65	2.00 [0.00, 4.00]	NA	NA
qSOFA	1.00 [0.00, 2.00]	NA	NA
**DISEASE COURSE**			
Duration of symptoms prior to admission (days)	4.00 [0.00, 30.0]	NA	NA
Hospital length of stay (days)	4.00 [0.00, 13.0]	NA	NA
28-day mortality	27 (100.0)	NA	NA

Continuous data are presented as mean (standard deviation) or median [interquartile range], and compared using a two-sided t-test or two-sided Wilcoxon rank-sum test, respectively. Categorical data are presented as count (percentage) and compared using Fisher's exact test. ACE = Angiotensin-converting enzyme, AT = Angiotensin, CAP = community-acquired pneumonia; CURB-65 = confusion, blood urea nitrogen, respiratory rate, blood pressure, age 65 or older; NA = not available or applicable, qSOFA = quick sequential organ failure assessment score.

Table S1C. Clinical characteristics and disease course in cohort with membrane expression of GLUT and 2-NBDG uptake in monocytes and neutrophils.

	CAP n=19	Controls n=19	*P* value
**DEMOGRAPHICS**			
Age (years)	67.4 (12.9)	59.6 (16.7)	0.12
Gender (male)	10 (55.6)	12 (63.2)	0.74
BMI	24.0 (4.65)	27.2 (7.65)	0.15
**COMORBIDITIES**			
Asthma	7 (36.8)	2 (10.5)	0.12
Chronic obstructive pulmonary disease	2 (10.5)	0 (0.0)	0.49
Diabetes mellitus, type 1	0 (0.0)	0 (0.0)	NA
Diabetes mellitus, type 2	2 (10.5)	3 (15.8)	>0.99
Hypertension	6 (31.6)	8 (42.1)	0.74
Myocardial infarction	4 (21.1)	0 (0.0)	0.11
Stroke	1 (5.3)	2 (10.5)	>0.99
Chronic kidney disease	2 (10.5)	0 (0.0)	0.49
**CHRONIC MEDICATION**			
Oral antidiabetics	1 (5.3)	3 (15.8)	0.60
Insulin	0 (0.0)	3 (15.8)	0.23
**LABORATORY TESTS**			
Thrombocytes (x10^9/L)	239 [101, 458]	244 [119, 693]	0.96
Leukocytes (x10^9/L)	10.3 [4.80, 30.5]	5.80 [4.40, 16.3]	0.006
Neutrophils (x10^9/L)	8.43 [3.60, 23.3]	3.71 [2.92, 10.2]	0.002
Lymphocytes (x10^9/L)	0.74 [0.40, 2.75]	1.23 [0.77, 3.84]	0.005
C-reactive protein (mg/L)	137 [3.10, 453]	1.40 [0.30, 31.6]	0.001
**DISEASE SEVERITY**			
Pneumonia Severity Index	4.00 [1.00, 5.00]	NA	NA
CURB-65	1.00 [0.00, 3.00]	NA	NA
qSOFA	0.00 [0.00, 1.00]	NA	NA
**DISEASE COURSE**			
Duration of symptoms prior to admission (days)	3.00 [1.00, 7.00]	NA	NA
Hospital length of stay (days)	5.00 [1.00, 23.0]	NA	NA
28-day mortality	16 (100.0)	NA	NA

Continuous data are presented as mean (standard deviation) or median [interquartile range], and compared using a two-sided t-test or two-sided Wilcoxon rank-sum test, respectively. Categorical data are presented as count (percentage) and compared using Fisher's exact test. ACE = Angiotensin-converting enzyme, AT = Angiotensin, CAP = community-acquired pneumonia; CURB-65 = confusion, blood urea nitrogen, respiratory rate, blood pressure, age 65 or older; NA = not available or applicable, qSOFA = quick sequential organ failure assessment score.

Table S2. Number of positive cultures in distant organs of myeloid cell-specific GLUT1 deficient mice and controls after infection with *S. pneumoniae* via the airways

	12 hours				42 hours		
	Control mice	*Slc2a1* – ΔM mice	P value		Control mice	*Slc2a1* – ΔM mice	P value
**Blood**	2 / 7	3 / 8	0.71		5 / 8	6 / 7	0.31
**Spleen**	2 / 7	7 / 8	**0.02**		7 / 8	6 / 7	0.92
**Liver**	3 / 7	2 / 8	0.59		7 /8	7 / 7	0.33

Number of positive cultures of distant organs from *Slc2a1*-ΔM and littermate controls 12 hours (left side) and 42 hours (right side) after intranasal inoculation with approximately $2x10^6$ CFUs of *S. pneumoniae*. Significance was calculated with a Chi-square test.

Table S3. Number of positive cultures in distant organs of granulocyte-specific GLUT1 deficient mice and controls after infection with *S. pneumoniae* via the airways

	12 hours				40 hours		
	Control mice	*Slc2a1* – ΔGr mice	P value		Control mice	*Slc2a1* – ΔGr mice	P value
**Blood**	1 / 7	1 / 9	0.85		2 / 8	5 / 8	0.13
**Spleen**	2 / 7	1 / 9	0.37		7 / 8	8 / 8	0.30
**Liver**	0 / 7	0 / 9	>0.99		5 / 8	8 / 8	0.05

Number of positive cultures of distant organs from *Slc2a1*-ΔGr and littermate controls 12 hours (left side) and 40 hours (right side) after intranasal inoculation with approximately $2x10^6$ CFUs of *S. pneumoniae*. Significance was calculated with a Chi-square test.

Table S4. Number of samples of control mice, myeloid cell-specific GLUT1 deficient mice and granulocyte-specific GLUT1 deficient mice

	Time point	Control mice	*Slc2a1* – ΔM mice		Time	Control mice	*Slc2a1* – ΔGr mice
**CFU**	12h<br>42h	7<br>8	8<br>7		12h<br>40h	7<br>8	9<br>8
**Pathology**	12h<br>42h	7<br>8	8<br>7		12h<br>40h	7<br>8	9<br>8
**MPO**	12h<br>42h	7<br>8	8<br>7		12h<br>40h	7<br>8	9<br>8
**TNF**	12h<br>42h	7<br>8	8<br>7		12h<br>40h	7<br>8	9<br>8
**IL-1β**	12h<br>42h	7<br>8	8<br>7		12h<br>40h	7<br>8	9<br>8
**IL-6**	12h<br>42h	7<br>8	8<br>7		12h<br>40h	7<br>8	9<br>8
**CXCL1**	12h<br>42h	7<br>7*	8<br>7		12h<br>40h	7<br>8	9<br>8
**CXCL2**	12h<br>42h	7<br>7*	8<br>7		12h<br>40h	7<br>8	9<br>8

* one sample was excluded as outlier.
